# Identification of novel small molecule inhibitors of ETS transcription factors

**DOI:** 10.1002/1873-3468.70040

**Published:** 2025-04-11

**Authors:** Shaima Abdalla, Zary Forghany, Jin Ma, Johan G. Hollander, Ruta Nachane, Karoly Szuhai, Pancras C. W. Hogendoorn, Peter ten Dijke, Dipen Shah, David A. Baker

**Affiliations:** ^1^ Oncode Institute and Department of Cell & Chemical Biology Leiden University Medical Center (LUMC) The Netherlands; ^2^ ZoBio BV Leiden The Netherlands; ^3^ Department of Cell & Chemical Biology Leiden University Medical Center (LUMC) The Netherlands; ^4^ Department of Pathology Leiden University Medical Center (LUMC) The Netherlands

**Keywords:** angiogenesis, cancer, drug development, ETS transcription factor

## Abstract

The evolutionarily conserved E‐Twenty‐Six (ETS) family of transcription factors acts downstream of major signal transduction pathways and plays a pivotal role in tissue development and maintenance. Importantly, their function is frequently corrupted in a substantial proportion of tumour types, and they are also indispensable for angiogenic sprouting, a hallmark of cancer, which is essential for fuelling tumour enlargement and dissemination. Consequently, targeting aberrant ETS activity could potentially represent a precise and effective means by which to block tumour growth. Here, we present proof‐of‐principle high‐throughput screens and an initial characterization of candidate hits, as a methodological and conceptual framework for the identification of novel ETS transcription factor inhibitors, which may ultimately lead to new therapeutic avenues for treating cancer.

## Graphical abstract

ETS transcription factors play an essential role in tumourigenesis and are indispensable for sprouting angiogenesis, a hallmark of cancer, which fuels tumour expansion and dissemination. Thus, targeting ETS transcription factor function could represent an effective, multifaceted strategy to block tumour growth.

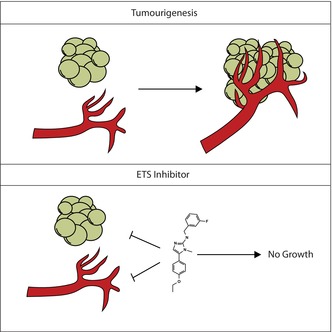

## Abbreviations

**AP1**, activator protein 1

**DLAV**, dorsal longitudinal anastomotic vessel

**DTT**, dithiothreitol

**EDBD**, ETS DNA‐binding domain

**ETS**, E‐twenty‐six

**GFP**, green fluorescent protein

**hiPSC‐CM**, human‐induced pluripotent stem cell‐derived cardiomyocytes

**HTRF**, homogeneous time‐resolved fluorescence

**HTS**, high‐throughput screen

**LCMS**, liquid chromatography mass spectrometry

**NMR**, nuclear magnetic resonance

**PBS**, phosphate buffered saline

**PECAM1**, platelet and endothelial cell adhesion molecule 1

**PH**, pleckstrin homology

**RFU**, relative fluorescence units

**STR**, short tandem repeat

**TERT**, telomerase reverse transcriptase

**TINS**, target immobilized NMR screening

**VEGF**, vascular endothelial growth factor

In the past two decades, a sea change in pharmacological approaches to cancer treatment has involved a shift away from traditional chemotherapies towards precision therapies directed against specific proteins in tumour cells (and the tumour stroma) with the aim of limiting cytotoxicity in healthy tissues [1, 2, 3, 4, 5]. While there have been improvements in patient outcomes, the promised revolution has thus far failed to materialize. Two of the principal reasons for this are the acquisition of resistance to treatment [6, 7, 8, 9, 10] and the fact that this category of inhibitors has, to date, targeted a relatively limited range of proteins comprising kinases and cell surface receptors [11, 12, 13]. One potential solution to these problems is to identify novel targets, particularly now that there is broader acceptance of the utility of searching beyond the historical constraints of the previously termed druggable genome (essentially composed of the aforementioned enzymes, and cell surface proteins) [14, 15].

In this context, Darnell [16] was correct to argue that there are a limited number of overactivated transcription factors in cancer, which could be suitable candidates for the development of anticancer drugs. Accordingly, there are compelling reasons to think that the ETS family of transcription factors could represent an excellent target since ETS family members could play a critical role in regulating many essential processes in cancer cell and tissue biology including cell cycle control, cell proliferation, cell differentiation and metastasis [17, 18, 19]. The founding, and prototypical, member of the family, ETS1, was first identified as an oncogenic fusion protein in the E26 avian leukaemia retrovirus [20, 21]. To date, 28 distinct ETS family members have been described in humans, which are classified into 12 subgroups based on protein domain sequence homologies [22]. Operationally, ETS proteins function by binding to consensus DNA sites (encompassing a core GGA(A/T) sequence) via a highly conserved ETS DNA‐binding domain (EDBD), an (approximately) 85 amino acid winged helix‐turn‐helix structure encoded by all family members [22, 23]. In the last 40 years, a substantial body of evidence has unveiled the importance of ETS factors in the evolution of numerous haematological and solid tumours [24, 25]. Multiple different mechanisms have been shown to underlie pathological ETS misregulation including chromosomal rearrangements, which generate ETS gene fusions in leukaemia, Ewing sarcoma, breast cancer, gastric cancer, head and neck cancer, and prostate cancer; gene amplification in breast cancer and melanoma; gain‐of‐function mechanisms linked to increased ETS factor activity and stability, for example, in glioblastoma, melanoma and bladder cancer [26]. Moreover, large‐scale genomic surveys identified mutations in the telomerase reverse transcriptase (TERT) gene, which encodes the catalytic reverse transcriptase subunit of telomerase, as one of the most prevalent genetic changes in a wide variety of tumours [27, 28]. While telomerase activity is usually absent in postembryonic somatic cells, it is reactivated in 90% of aggressive cancers through the upregulation of TERT [29, 30]. Two mutually exclusive mutations close to the transcription start site, which create *de novo* ETS binding sites and putatively lead to aberrant binding of specific ETS factors, are believed to underlie the tumour‐promoting upregulation of TERT activity [27, 28]. Importantly, ETS transcription factors are also indispensable for angiogenic sprouting [31, 32, 33], including the process by which tumours stimulate the expansion of the local vasculature, which physically connects them to the circulation, thereby fuelling tumour growth and dissemination.

Given the sound rationale for targeting ETS proteins as a potential anticancer therapy [34, 35], in recent years, computational chemistry methods and phenotypic and functional screens have been deployed to discover ETS inhibitors [36, 37, 38, 39, 40, 41, 42]. However, there are technical challenges that must be surmounted to identify specific inhibitors that directly block ETS activity, not least because transcription factors are enriched with intrinsically disordered regions and lack obvious, well‐defined binding pockets [43, 44], which explains the paucity of successful small molecule screening campaigns to date. Despite such possible constraints, the EDBD is potentially sufficiently structured to interact with small molecules, and since ETS activity is largely dependent on DNA‐binding via the EDBD, this domain could represent a tractable target. In this study, we utilized optimized methodologies to target the EDBD. Two main strategies have been deployed. First, we used target immobilized nuclear magnetic resonance (NMR) screening (TINS) [45] to identify small molecular scaffolds that can bind directly to the EDBD. Given the relatively smaller scale, such proof‐of‐principle approaches have been used routinely by the pharmaceutical/biotechnology industry as an initial step to test the potential ligandability of target proteins [46, 47]. The second approach employed a novel multicomponent high‐throughput functional screen of small molecules, which disrupt ETS binding to a consensus DNA‐binding site.

Our work suggests that high‐throughput screening approaches could be used to identify specific small molecule inhibitors of ETS transcription factors.

## Materials and methods

### Protein expression and purification

C‐terminal His_6_ epitope‐tagged EDBDs were cloned into pET28a and expressed in Rosetta DE3 *Escherichia coli*. Harvested cells were resuspended in lysis buffer [500 mm NaCl, 20 mm imidazole, 2 mm DTT, 10% glycerol, 1× phosphate buffered saline (PBS), pH 7.4] on ice and sonicated (30 s on, 30 s off) for 5 min. Lysates were centrifuged at 24 500 x ***g*** for 40 min at 4 °C. Supernatants were filtered through low protein binding 0.22‐μm filters (Merck Millipore, Tullagreen, Cork, Ireland), loaded onto a 5 mL HisTrap HP column (Cytiva, Uppsala, Sweden) and eluted with a gradient in elution buffer [500 mm NaCl, 300 mm imidazole, 2 mm dithiothreitol (DTT), 10% glycerol, 1× PBS, pH 7.4]. Pooled fractions were diluted 10 times in heparin column buffer (20 mm HEPES, 1 mm DTT, pH 7.4), loaded onto a 5 mL HiTrap Heparin column (Cytiva) and eluted with a gradient in Heparin Buffer B: 1 m NaCl, 20 mm HEPES, 1 mm DTT, pH 7.4. Protein fractions were concentrated using Amicon‐Ultra Centrifugal Filter (Merck Millipore), loaded onto a gel filtration Superdex 200 16/600 column (Cytiva) and equilibrated in buffer (150 mm NaCl, 1 mm DTT, 1× PBS, pH 7.4). Fractions of each step were run on 12% SDS/PAGE gels and stained with Colloidal Blue Staining Kit Invitrogen (Thermo Fisher Scientific, Carlsbad, CA, USA).

### Target immobilized NMR screening (TINS)

Purified C‐terminal His_6_ epitope‐tagged ETV6 DBD [amino acids 1–118 (PDB 2DAO numbering) corresponding to amino acids 334–452] was immobilized via amine coupling to 600 μL Actigel‐ALD resin (Sterogene, Carlsbad, CA, USA) in 25 mm HEPES pH 7.5, 100 mm NaCl and 2 mm MgCl_2_ at 4 °C (following the manufacturer's protocol). The reference protein [the pleckstrin homology (PH) domain of Akt, which represents a typical protein surface but is devoid of any specific small molecule binding sites] was immobilized using the same protocol. The immobilization efficiency was above 90%, and the final concentrations of the immobilized targets were in the 100 μm range. TINS experiments were performed on a 500 MHz Bruker NMR spectrometer using spatially selective Hadamard pulse sequences and analysed as described previously [46, 47, 48, 49]. The fragment library consisted of 1364 compounds with an average molecular weight of ~ 200 Da and complied with the general rules of 3 (molecular weight of a fragment is < 300, the cLogP is ≤ 3, the number of hydrogen bond donors is ≤ 3 and the number of hydrogen bond acceptors is ≤ 3, number of rotatable bonds is ≤ 3). The library exhibited significant structural diversity, encompassing a wide range of functional groups, stereochemical configurations and distinct scaffolds. The library was optimized for screening a highly diverse chemical space and included aromatic, heterocyclic and aliphatic frameworks with functional groups such as sulfonamides, amides, alcohols, ketones and halides. The fragment mixes, containing 2–8 compounds per mix, were prepared by mixing their d6DMSO stocks (at 100 mm) and further dilution into NMR‐buffer (deuterated PBS) to a final concentration of 500 μm. The mixes have been carefully designed based on individual 1D 1H NMR spectra of the fragments to minimize spectral overlap and to avoid solubility issues. Screening was performed by repeated cycles of injection of fragment mixes into both the cells of a dual‐cell packed with immobilized EDBD and the reference protein. Following injection of a mix, the flow was stopped and the NMR data were acquired. The fragments were washed out prior to the subsequent injections. The binding of a fragment to the EDBD could be detected by a simple reduction in the height of the NMR signals from that fragment in the presence of the target protein relative to the peak intensities in the presence of the reference protein. Preferential binding of the fragments to the target (T) and reference (R) proteins (defined as the T/R value) was determined using the signal amplitudes of a compound in the presence of the target divided by the signal amplitudes of the compound in the presence of the reference (T/R value). A cut‐off T/R value of 0.5 was chosen for the selection of hit candidates. The fragment screen identified 103 unique hit candidates (a hit rate of 7.5%).

### High‐throughput small molecule screen

The homogeneous time‐resolved fluorescence (HTRF) assay is depicted schematically in Fig. 3 and described in the text. The screen was performed in a 1536‐well (Corning, Kennebunk, ME, USA) format (4 μL end volume) using a BioRAPTR (Beckman Coulter, Brea, CA, USA). The following conditions were stringently optimized: 40 nm protein/4 nm Eu‐anti‐His antibody (Cisbio Bioassays, Codolet, France); 62.5 nm Strep‐XL665 (Cisbio)/62.5 nm biotinylated oligo ±500 nm nonbiotinylated oligo. Compounds were tested at a concentration of 10 μm (*Z*′ score = 0.76; range 0.57–0.85). The nonbiotinylated oligo competitor was considered to be maximally inhibitory, and a cut‐off of 50% inhibition was used in the primary assay to select potential hit compounds. Approximately 400 000 compounds were screened, of which 398 892 could be validated and verified. A total of 2706 compounds were selected for further validation. A total of 277 compounds could be confirmed in an independent repeat of the screen, of which 83 compounds gave > 50% inhibition and were selected for further characterization. Potency and efficacy were assessed using dose–response curves with seven concentrations (range 20 μm to 20 nm, *n* = 2), with the selection criteria set at a pEC50 > 5. A deselection assay using the DNA‐binding domain of SMAD3 was employed to remove compounds exhibiting nonspecific activity. Lastly, cheminformatic analysis using Bayesian models helped refine the list and further eliminate possible false positives.

### NMR backbone sequence assignment

*Escherichia coli* BL21 (DE3) expressing His_6_ epitope‐tagged ETV6 EDBD was cultured in M9 minimal medium supplemented with ^15^NH_4_Cl and ^13^C‐d‐Glucose (CIL) as a sole nitrogen and carbon source. The labelled protein was purified as described above. The sequential backbone assignments were obtained by correlation of Cα and Cβ chemical shifts of i and i‐1 residues to the amide ^1^H and ^15^N resonances using HNCACB, CBCAcoNH, HNCA and HNcoCA spectra acquired at 296 K on a 600 MHz BRUKER DMX NMR spectrometer equipped with a TXI cryoprobe. The acquired data were processed using nmrpipe and visualized on Sparky [50, 51]. The assignment process was guided by the predicted chemical shifts calculated by SHIFTX in the automatic assignment program MARS using the PDB‐2DAO structure as a reference [52, 53].

### Chemical shift perturbation analyses

The [^1^H,^15^N]‐HSQC spectra were acquired at 296 K on a 600 MHz Bruker DMX NMR spectrometer equipped with a TXI cryoprobe. The NMR sample was prepared in 25 mm HEPES pH 7.5, 100 mm NaCl, 1 mm EDTA and 1 mm β‐mercaptoethanol. The typical NMR sample contained 0.130 mm of the protein and fragments at various concentrations (see Results section) and 5% d6‐DMSO. The pH of the samples was adjusted carefully within ±0.05 units after addition of the compound. A total of 128 indirect increments with 16 scans per increment were acquired. The data were processed using Topspin 1.2/2.1 (Bruker, Billerica, MA, USA) and visualized on Sparky [51]. Chemical shift perturbations in [^1^H,^15^N]‐HSQC spectra were calculated based on the change in ^1^H and ^15^N ppm value in the presence and absence of a compound (Δδ > two times standard deviation + Δδavg). The potential binding sites of a compound were mapped onto the surface of the ETV6 EDBD (PDB‐2DAO) structure using chemical shift perturbation data and viewed in pymol (Schrödinger, New York, NY, USA).

### Surface plasmon resonance studies

DNA binding to the ETV6 EDBD was tested using surface plasmon resonance on a T200 biacore instrument (Cytiva). pH scouting was performed to determine the optimum pH for protein immobilization on the CM5 chip surface. Following immobilization of the protein (6000 RU), increasing concentrations of DNA were titrated in a single‐cycle kinetic mode. The buffer conditions were as follows: 25 mm HEPES pH 7.5, 100 mm NaCl, 1 mm EDTA and 1 mm β‐mercaptoethanol. The analysis was performed using a biacore evaluation software (Cytiva) and graphpad prism (version 10.2. 3; GraphPad Software, San Diego, CA, USA).

### Cell culture, biochemistry

The prostate cancer cell line, VCaP (RRID: CVCL_2235), was cultured in Iscove's Modified Dulbecco's Medium (IMDM) (Gibco, Grand Island, NY, USA), supplemented with 15% Fetal Bovine Serum (FBS) (Gibco). SK‐ES‐1 Ewing sarcoma cells (RRID: CVCL_0627) were cultured in IMDM supplemented with 10% FBS. Primary HUVECs (Lonza, Basel, Switzerland) were cultured in EGM2 medium (Lonza). MDA‐MB‐231 cells (RRID: CVCL_0062) were cultured in Dulbecco's Modified Eagle Medium (DMEM) (Gibco) supplemented with 10% fetal bovine serum (FBS) (Gibco). All cell lines were obtained from the American Type Culture Collection (ATCC, Manassas, VA, USA). Cells were maintained in a 5% CO_2_, 37 °C humidified incubator, tested monthly for mycoplasma contamination and checked for authenticity in the last 3 years by an in‐house service using short tandem repeat (STR) profiling.

### Proliferation assays

Cells were seeded, in triplicate, into 96‐well white plates with a clear flat bottom in 100 μL of medium at the following initial densities: VCaP, 30 000 cells·mL^−1^; HUVECs and SK‐ES‐1, 40 000 cells·mL^−1^. The medium was supplemented with the indicated concentrations of compounds (see Results section). The number of viable cells was determined using a Cell Titer‐Blue reagent (Promega, Madison, WI, USA) at 0, 3, and 5 days after treatment. Absorbance readings were taken at 544 nm/590 nm relative fluorescence units (RFU) using the VICTOR X3 Multilabel Plate Reader (PerkinElmer, Waltham, MA, USA).

### *In vitro* DNA‐binding assay

*In vitro* translated protein was made using the TNT‐coupled reticulocyte *in vitro* transcription/translation system (Promega). 50 pmol of biotinylated double‐stranded oligonucleotides harbouring either three consensus ETS binding sites (for the ETV6, FLI1, ERG transcription factors) or three activator protein‐1 (AP‐1) binding sites (for JUN) were coupled to Dynabeads™ MyOne™ Streptavidin C1 beads (Invitrogen, Thermo Fisher Scientific). Double‐stranded oligonucleotides were incubated with *in vitro* translated proteins in the presence or absence of compounds at concentrations highlighted in the figure legends. Reactions were incubated at 4 °C, with shaking, for 30 min in the presence of 1 μg of poly (dI/dC), 4 mm spermidine. Beads were successively washed ×4 with binding buffer (50 mm KCl, 10 mm HEPES (pH 7.6), 5 mm MgCl_2_, 10 mm Tris (pH 8), 0.05 mm EDTA (pH 8), 0.05 mm, 0.1% Triton X‐100, 20% glycerol). Associated proteins were eluted in Laemmli buffer, and protein–DNA interactions were determined by western blotting using a FLAG mouse monoclonal antibody (Sigma‐Aldrich, St. Louis, MO, USA). The following oligonucleotide sequences were used:

ETS 5′ ACCGGAAGTACCGGAAGTACCGGAAGT 3′
AP1 5′ TGACTCATGAGTCAGTATGAGTCACAATGACTCACCT 3′

### Zebrafish angiogenesis assay

The experiments were conducted in a licensed establishment for the breeding and use of experimental animals (Leiden University) and subject to internal regulations and guidelines, stating that advice is taken from the animal welfare body to minimize suffering for all experimental animals housed at the facility. The zebrafish assays described are not considered an animal experiment under the Experiments on Animals Act (Wod, effective 2014), the applicable legislation in the Netherlands in accordance with the European guidelines (EU directive no. 2010/63/EU) regarding the protection of animals used for scientific purposes, because noneating larvae were used. Therefore, a license specific for these assays on zebrafish larvae (< 5 days) was not required. The *fli1a:gfp* transgenic line produces embryos in which all of the endothelial cells are marked by GFP. Coupled to their optical transparency, this enables direct visualization of angiogenesis. During the first 2 days of development, the reiterated pattern of intersegmental trunk vessels (ISV) is formed by angiogenic sprouts from dorsal aorta endothelial cells that grow to the dorsal side of the trunk where they interconnect to form the dorsal longitudinal anastomotic vessel (DLAV). For the experiments described here, embryos were kept in egg water (60 μg·mL^−1^ sea salts; ~ 60 embryos/dish) at 28 °C. Following fertilization, drug treatments were added directly to the egg water. After 24 h, chorions were removed mechanically using forceps. Embryos were anaesthetized using tricaine methanesulfonate (Sigma‐Aldrich) at a final concentration of 0.003% in egg water, approximately 10 min prior to imaging. Imaging of vessels was carried out by using a Leica SP5 confocal microscope (Leica Microsystems, Wetzlar, Germany) using a ×10 or ×20 objective. Vessel characteristics of 20 embryos per condition were scored.

### Metatarsal assay

Embryos were isolated from the uterus and kept in PBS on ice. The metatarsal dissection procedure [54] was performed on mouse embryos at Day 17 of gestation. Metatarsals were cultured, in the presence or absence of drug treatments, at 37 °C in 0.1% gelatin‐coated dishes in MEM Alpha Medium (Gibco) and GlutaMAX (Gibco), which contained 10% FBS (Gibco) and penicillin–streptomycin (ICN Biomedicals, Aurora, OH, USA). This medium was supplemented with 50 ng·mL^−1^ recombinant human VEGF 165 (R&D Systems, Minneapolis, MN, USA). The culture medium was changed every 2 days. Following 5 days of culture, vessel outgrowth from metatarsals was visualized by immunostaining. Metatarsals were washed with DPBS (Dulbecco's PBS; Gibco) and fixed in Zink Macrodex Formalin (PFA; Sigma‐Aldrich) for 15 min. Staining of the metatarsals was performed using the CD31 antibody (BD Biosciences, San Diego, CA, USA) as previously described [55]. For a detailed analysis of vessel formation, vessel configurations were converted into black‐and‐white binary images. Vascular area (black) in pixels was quantified using in‐house computer software developed by the Department of Cell and Chemical Biology at the LUMC. In all the treatments, the total vascular area has been normalized against the area encompassing the metatarsal bones [54]. The mice experiments were approved by the Institutional Committee for Animal Welfare of the Leiden University Medical Center (LUMC) and were performed according to the regulatory guidelines.

### Cardiomyocytes

The human‐induced pluripotent stem cell‐derived cardiomyocytes (hiPSC‐CM) (provided by R. Davis, LUMC) were grown on 5‐mm coverslips in 100 μL Matrigel™ (Corning) and 1 mL culture medium [DMEM (Gibco) supplemented with 10% FBS (Gibco), 450 μm α‐MTG, 0.05 mg·mL^−1^ l‐ascorbic acid 2‐phosphate (Sigma‐Aldrich), 2 mm GlutaMAX (Gibco), 0.5% Penicillin/streptomycin (ICN Biomedicals)]. Cells were incubated at 37 °C in the presence or absence of the indicated compounds (Fig. 4D). Following 16 h of treatment, the cells were fixed with 4% paraformaldehyde‐phosphate buffered saline (PBS) for 15 min and permeabilized in 0.2% Triton X‐100‐PBS for 5 min. Subsequently, the cells were washed with PBS 0.5% Tween and blocked with a solution containing PBS/0.5% Tween and 5% BSA for 30 min. Immunostaining was performed with the primary antibodies, Troponin I (Santa Cruz Biotechnology, Santa Cruz, CA, USA) and anti‐phospho‐histone H2A.X (Ser139) (Cell Signaling Technology, Danvers, MA, USA), as previously described [55]. Cells were imaged using a Leica SP8 confocal microscope.

### Antibodies

The following primary antibodies were used: FLAG M2 mouse monoclonal (Sigma‐Aldrich), anti‐HA.11 mouse monoclonal (Covance, Princeton, NJ, USA), anti‐HA rabbit polyclonal (Abcam, Cambridge, UK), anti‐Troponin (Santa Cruz Biotechnology), anti‐phospho‐histone H2A.X (Ser139) (Cell Signaling Technology). The following secondary antibodies were used: anti‐Rabbit Alexa Fluor® 488 and anti‐Mouse Alexa Fluor® 594 (Thermo Fisher Scientific).

## Results

### A screen [target immobilized NMR screening (TINS)] to identify novel molecular fragment binders to the ETS DNA‐binding domain (EDBD)

**Fig. 1:**
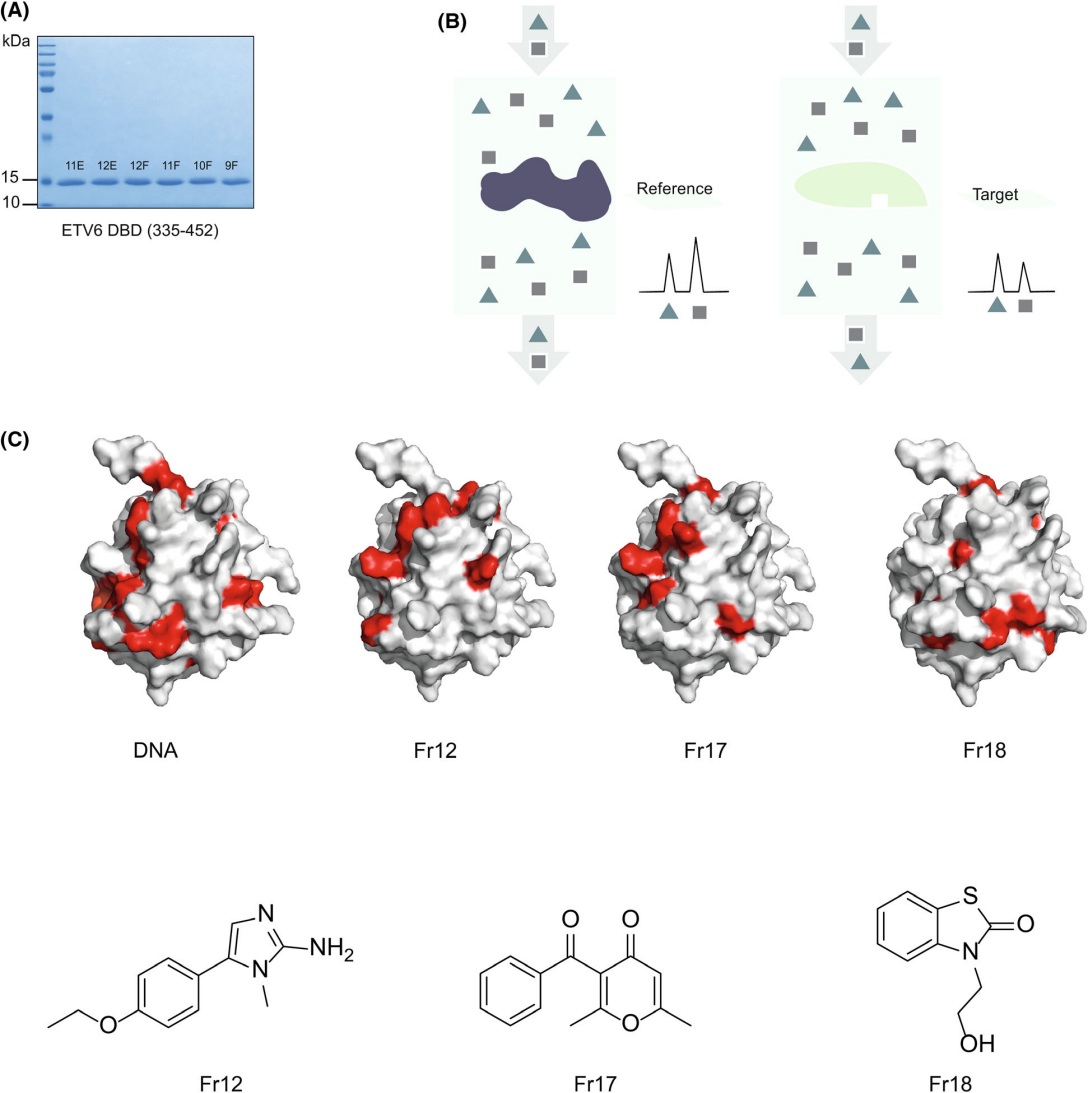
A fragment library screen to identify molecular interactors with the EDBD. (A) Coomassie stain of the ETV6 EDBD following purification via nickel column and heparin column chromatography followed by size‐exclusion chromatography on an S200 16/60 column. Purified protein in the indicated elution fractions is shown (see Materials and methods section). (B) Schematic representation of TINS (see Materials and methods section). Briefly, a fragment library was screened by simultaneously injecting a mix of fragments into a dual‐cell sample holder with immobilized ETV6 EDBD as a target, and the reference protein. The binding of a fragment to the EDBD could be detected by a simple reduction in the height of the NMR signals from that fragment relative to the reference protein [45]. (C) Validation of the candidate hit fragments by NMR chemical shift mapping of the EDBD residues upon fragment binding. The EDBD‐DNA‐binding interface is compared to EDBD‐fragment binding. The chemical structures of the selected hit fragments is shown.

Since ETS factors mediate their biological effects by binding to consensus DNA‐binding sites via their highly conserved EDBDs, as a first step towards validating the EDBD as a potentially suitable target for the identification of specific small molecule inhibitors of ETS factor function, we optimized purification protocols to produce milligram quantities of pure recombinant EDBD (Fig. 1A), which could functionally bind to an oligonucleotide harbouring 3× consensus ETS DNA‐binding sites when immobilized under screening conditions [determined by surface plasmon resonance (SPR) and subsequently confirmed by NMR (Fig. 1C)].

To identify molecules that could directly interact with the EDBD, initially, we screened a fragment library using TINS (schematically represented in Fig. 1B), an NMR‐based approach that is optimal for identifying low‐affinity hit–target interactions [45]. These small molecular scaffolds (with a mean mass of approximately 200 Daltons) enable a broader exploration of biochemical interfaces and provide a preliminary quantitative measure of target ligandability [45, 46]. A total of 1364 commercially available fragments were screened, yielding 103 unique hit candidates. A selection of the hits with relatively higher affinities for the EDBD target was chosen for further characterization. First, we deployed protein‐observed NMR to map the site of binding of hits to the EDBD (Fig. 1C). Sequential EDBD backbone assignments were obtained by correlation of Cα and Cβ chemical shifts of i and i‐1 residues to the amide ^1^H and^15^N resonances using HNCACB, CBCAcoNH, HNCA and HNcoCA spectra (see Materials and methods section). Notably, the pattern of contact between the EDBD and a consensus DNA site potentially overlaps with the points of contact between the EDBD and a subset of hit compounds, suggesting that such molecules or analogues of the molecules might disrupt ETS factor binding to DNA.

### Validation of fragment binding to the EDBD

**Fig. 2:**
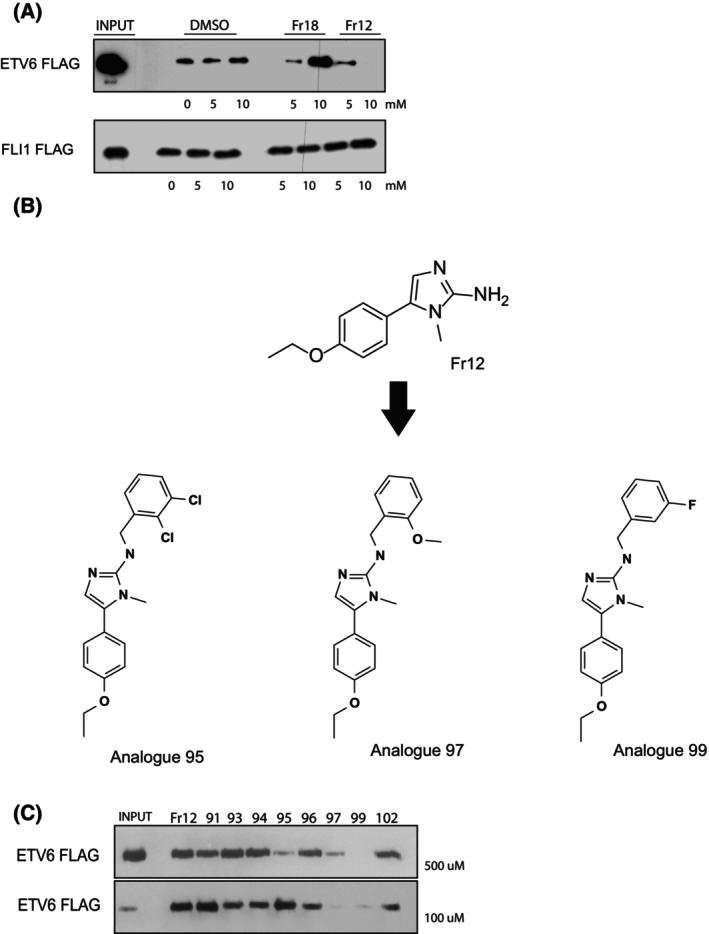
(A) A biotinylated oligonucleotide bearing 3× consensus ETS DNA‐binding sites was incubated with *in vitro* translated full‐length epitope‐tagged ETV6 protein or FLI1 protein in the presence or absence of the indicated concentrations of fragment hits. Protein‐DNA binding was visualized by Western blotting with the indicated antibody. Control DMSO concentrations corresponded to the final DMSO concentrations of the test compound. (B) The chemical structures of small molecule analogues of hit fragment Fr12 (see A), which were tested in C. (C) A biotinylated oligonucleotide bearing 3× consensus ETS DNA‐binding sites was incubated with *in vitro* translated full‐length epitope‐tagged ETV6 protein in the presence or absence of the indicated concentrations of chemical analogues of fragment Fr12. Protein‐DNA binding was visualized by Western blotting with an anti‐FLAG antibody.

To further characterize the hit fragments, we established an *in vitro* assay employing a biotinylated consensus ETS DNA‐binding site and *in vitro* translated ETS proteins to recapitulate the binding of ETS factors to DNA. This optimized assay was used to assess the effect of the fragments on ETS protein binding to its DNA‐binding site. Figure 2A illustrates at least two distinct classes of hits: one fragment (Fr18) enhanced ETS DNA binding, whereas a different fragment (Fr12) significantly inhibited ETS DNA binding. Interestingly, the fragments had no detectable effect on the binding of a related ETS factor to the consensus DNA site (Fig. 2A), suggesting that the fragments may exhibit a degree of specificity for their target. The very low affinities of the fragments for the target (in the mm range) were not unexpected owing to their relatively small size, and despite the evidence for a direct interaction between the fragment hits and the EDBD, the affinities would be too low to elicit biologically meaningful effects. To address this issue, we tested analogues of the hits, which share an identical core structure with the original fragment hit, and additional unique chemical side chains (Fig. 2B). Figure 2C shows that a subset of fragment analogues inhibited ETS DNA binding at significantly lower concentrations (500–100 μm, a 100‐fold increase in potency), by comparison with the fragment hit, in a functional *in vitro* binding assay.

Collectively, these data suggest that fragment‐based screening could potentially identify molecular scaffolds that bind to the EDBD, and subsequent optimization of the analogues might potentially generate compounds with superior affinities for the EDBD target protein compared to the original fragment hits. This approach has been routinely adopted by industry as a proof‐of‐principle test of the suitability of targets for larger scale screens [46]. In this context, we next performed a high‐throughput screen of 400 000 compounds.

### High‐throughput screen (HTS) to identify novel ETS factor inhibitors

**Fig. 3:**
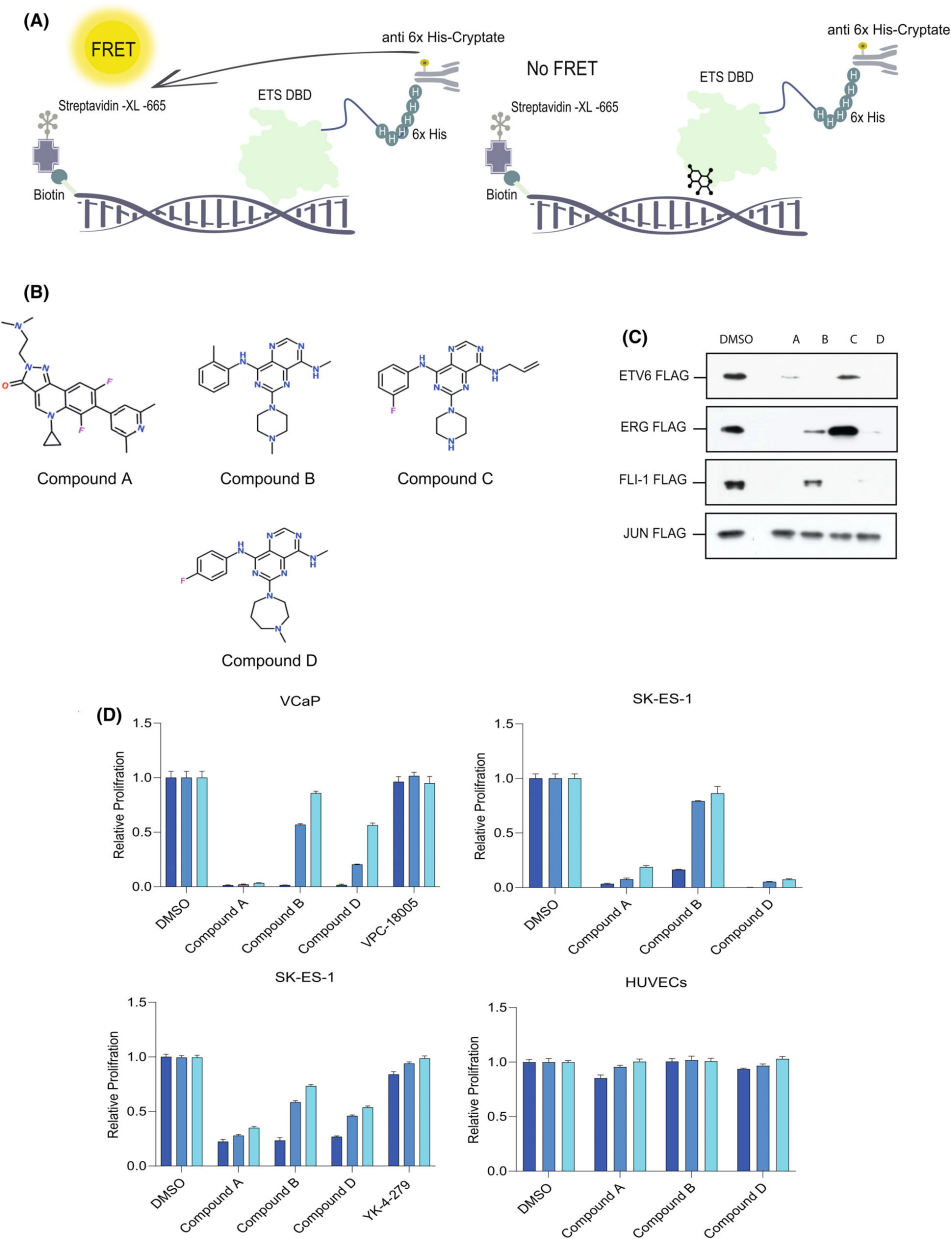
(A) Schematic representation of the HTS. A FRET signal was generated when the fluorophore conjugated to the anti‐His_6_ antibody (which recognized the His tag of the EDBD) was in close proximity to the XL‐665 streptavidin (which recognized the biotin moiety of the oligonucleotide). Compounds that could disrupt the binding of the ETS protein to the DNA site triggered a loss of the fluorescence signal. Using this assay, 400 000 small molecules were screened in the primary assay. (B) Chemical structures of the candidate hit compounds. (C) A biotinylated oligonucleotide bearing 3× consensus ETS DNA‐binding sites was incubated with the indicated *in vitro* translated full‐length epitope‐tagged ETS proteins in the presence or absence of 20 μm of the candidate hit compounds. *In vitro* translated epitope‐tagged JUN binding to a biotinylated oligonucleotide bearing 3× consensus AP‐1 DNA‐binding sites acted as a control. Protein‐DNA binding was visualized by Western blotting with an anti‐FLAG antibody. (D) Cell viability assays of the indicated tumour cell lines (see Materials and methods section), and control human umbilical vein endothelial cells (HUVECs), were performed in the presence or absence of the indicated treatments (5, 2.5, and 1 μm) for 5 days. VCaP cells encode an ERG fusion, and SK‐ES‐1 cells encode a FLI1 fusion. For comparison, the VPC‐18005 inhibitor, which has been reported to target the EDBD of the ERG protein [36], and the YK‐4‐279 inhibitor, which targets protein–protein interactions between the EWS‐FLI1 fusion protein and RNA helicase A [37], are included. Values represent ± SD of three replicates per data point.

Broadly, two types of small molecule screens can be performed to identify hits that could potentially inhibit the activity of a specific target protein: single component direct binding screens, such as the TINS screen described in Figs 1 and 2; and multicomponent functional screens in which small molecules disrupt a particular biochemical process, for example, protein–protein or protein‐DNA interactions. The assays must be rapid, scalable and automated. Here, we deployed a functional assay to quantifiably measure EDBD binding to a consensus DNA‐binding site. For this purpose, we employed Homogeneous Time‐Resolved Fluorescence (HTRF) [56, 57] to assay the binding of the His_6_ epitope‐tagged EDBD to a biotinylated oligonucleotide harboring three consensus ETS binding sites (see Fig. 3A; Materials and methods section). In brief, a FRET signal was generated when the fluorophore conjugated to the anti‐His_6_ antibody (which recognized the His tag of the EDBD) was in close proximity to the XL‐665 streptavidin (which recognized the biotin moiety of the oligonucleotide). Compounds that could disrupt the binding of the ETS protein to the DNA site caused a loss of the fluorescence signal (Fig. 3A). Using this assay, 400 000 small molecules were screened in the primary assay. Further validation to assess reproducibility (see Materials and methods section), coupled with liquid chromatography mass spectrometry (LCMS) analyses of the compounds, led to the selection of four hits, which measurably inhibited EDBD DNA binding, for simplicity named compounds A–D. The chemical structures of the hit candidates are depicted in Fig. 3B.

### Biochemical validation of hit compounds in functional *in vitro* binding assays

Figure 3C shows that the hit compounds efficiently inhibited ETS factor binding to its DNA consensus binding site but failed to inhibit JUN DNA binding to a related but distinct consensus DNA site, consistent with the idea that the inhibition is EDBD DNA binding specific. In addition to ETV6, the hit compounds also inhibited DNA binding of other ETS factors, including ERG and FLI1, suggesting that the inhibition is not limited to a specific ETS transcription factor, which presumably reflects the high degree of overall EDBD structural similarity within the ETS transcription factor family.

### Hit compounds significantly inhibit tumour cell proliferation

Since the hit compounds inhibited the binding to DNA of multiple different ETS transcription factors, we next tested the effects of the compounds on the proliferation of tumour cell lines. Multiple cancer cell lines (melanoma, pancreatic carcinoma, prostate carcinoma and Ewing sarcoma), in which ETS function is known to be corrupted, were tested, and comparable results were obtained. Figure 3D presents representative data on two cell lines, which harbor chromosome translocations resulting in aberrant FLI1 or ERG function in Ewing sarcoma [58] and prostate carcinoma cells [59, 60], respectively. The proliferation of the cancer cell lines was significantly suppressed by compounds A, B and D, each showing half‐maximal inhibitory concentration (IC50) values in the 1–10 μm range (Fig. 3D). By contrast, the compounds did not significantly block the proliferation of normal primary HUVECs to the same degree as the tumour cells.

### Hit compounds inhibit angiogenic sprouting

**Fig. 4:**
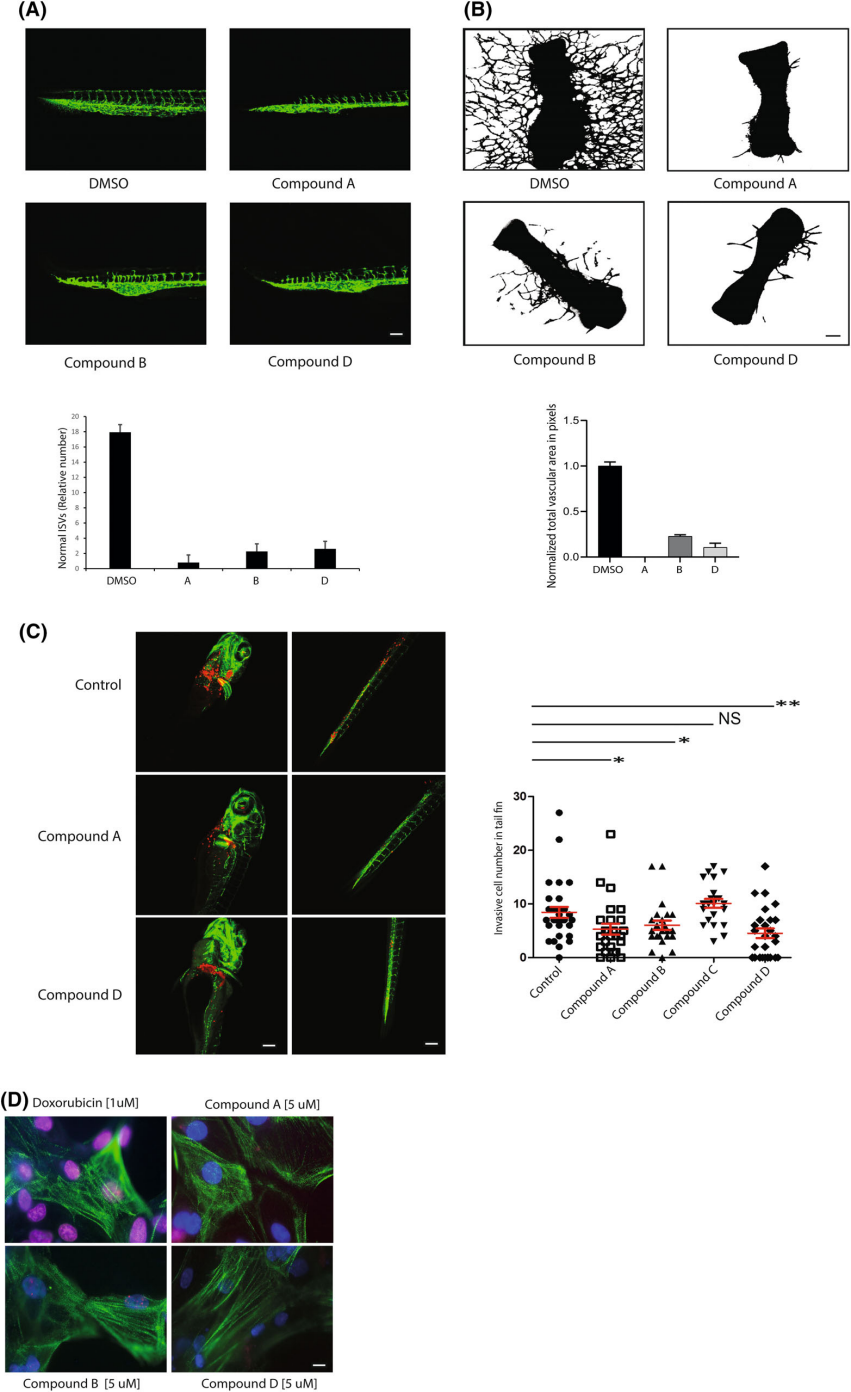
(A) Fli1a:gfp transgenic zebrafish embryos were treated with 10 μm of the indicated compounds for 16 h. Blood vessels were imaged by confocal microscopy, and disruption of the formation of the intersegmental vessels was quantified (see Materials and methods section). Scale bar = 150 μm. (B) Mouse metatarsal assay. Metatarsals isolated from fetal mice were incubated *ex vivo* in a defined vascular endothelial growth factor (VEGF) medium in the presence or absence of compounds (1 μm). Blood vessels were visualized using a CD31 antibody. Ten metatarsals were scored per condition (see Materials and methods section). Error bars represent the standard deviation of the mean. Scale bar = 150 μm. (C) Zebrafish embryo xenotransplant assay. Four hundred mCherry fluorescently labelled human breast cancer MDA‐MB‐231 cells were microinjected into the duct of Cuvier of 3‐day‐old zebrafish embryos. Tumour cell invasion into avascular tissue was measured 3 days later (see Materials and methods section). Shown is a representative experiment in which 25 embryos were scored per condition. The experiment was performed three times. Error bars represent the standard deviation of the mean. Left panels: Scale bar = 500 μm; Right panels: Scale bar = 150 μm. (D) Evaluating DNA Damage in hiPSC‐derived cardiomyocytes. hiPSC‐derived cardiomyocytes were cultured overnight in the indicated conditions. Shown are confocal images of cells stained for troponin I (green) and phospho‐histone H2A.X (red to visualize DNA damage). Nuclei were stained with DAPI (blue). Scale bar = 5 μm.

In addition to controlling the proliferation of tumour cells directly, ETS factors also play multiple other distinct roles in the biology of the tumour stroma, most notably in the tumour vasculature, since ETS factors are indispensable for the expansion of blood vessels via the process of angiogenesis [31, 32, 33]. In this light, we tested the effects of the compounds in three different angiogenesis assays. First, we used the *fli1*a:gfp transgenic zebrafish line that produces embryos in which all of the blood vessels are marked by GFP, which, coupled to the optically transparent nature of the embryos, enables systematic and dynamic visualization of angiogenesis. Figure 4A shows that incubation of zebrafish embryos with 10 μm of compounds A, B and D for 16 h resulted in clear disruption of angiogenesis, manifested by a reduction in the number of vessels, aberrant vessel trajectories and the premature stalling of dorsal aorta sprouts (Fig. 4A). Effects were most obvious for compounds A, B and D (Fig. 4A), whilst Incubation with drug C did not significantly disrupt vessel sprouting. These effects were not associated with overt, generalized toxicity.

To further characterize the effect of the small molecules on angiogenesis, we employed an *ex vivo* fetal mouse metatarsal assay, which recapitulates many of the critical features of angiogenesis observed *in vivo* [54]. Metatarsals were isolated from fetuses at embryonic stages E17.5 and incubated in a defined medium containing vascular endothelial growth factor (VEGF) in the presence or absence of 1 μm of the compounds. Vessel formation was monitored by phase‐contrast light microscopy from Day 2. Compounds A, B and D each inhibited ectopic angiogenesis of the cultured metatarsals. Immunofluorescence staining with platelet and endothelial cell adhesion molecule 1 (PECAM‐1) (CD31 antibody) was performed to visualize metatarsal vessel outgrowth. Confocal images of microvessels are shown (Fig. 4B). A quantitative analysis of vessel branching was performed based on the number of pixels in the vessel area (Fig. 4B). Figure 3D showed that the HTS hit candidates effectively inhibited tumour cell proliferation; however, the compounds did not significantly block endothelial cell proliferation, which express normal levels of ETS protein activity, to the same extent as the tumour cells, consistent with the idea that the observed effects of the compounds on angiogenesis principally result from inhibition of endothelial sprouting as opposed to endothelial cell proliferation.

Given the importance of ETS factors in cell proliferation and movement, coupled to their role in sprouting angiogenesis, an ETS inhibitor might suppress tumour cell invasion and metastasis. Here, we employed a Tg (*fli1*:gfp) zebrafish xenotransplantation assay to investigate cell extravasation and intravasation. Approximately 400 human breast cancer MDA‐MB‐231 cells (genetically labelled with mCherry) were injected into the duct of Cuvier 48 h after fertilization. Injected zebrafish embryos were treated with compounds by addition to culture water. Figure 4C shows that the dissemination of the breast cancer cells towards the head and tail was significantly reduced in the presence of compounds A, B and D (10 μm).

Taken together, these data suggest that, in addition to directly inhibiting the proliferation of tumour cells, selected hit compounds exert an inhibitory effect on angiogenic sprouting and tumour cell intravasation.

### Evaluation of hit compound cardiotoxicity

Short‐ and long‐term toxicity is a fundamental problem associated with chemotherapy treatments, in particular treatments that damage DNA. By example, doxorubicin, a widely used chemotherapy reagent, activates the DNA damage response machinery ultimately resulting in apoptosis and associated long‐term cardiotoxicity, which leads to cardiac tissue damage and potential heart failure [61]. Therefore, cardiotoxicity is one of the most important considerations when developing novel therapies with the aim of improving current treatments. Here, we used human‐induced pluripotent stem cell‐derived cardiomyocytes (hiPSC‐CM) to evaluate cardiotoxicity of the hit compounds, utilizing an antibody targeting phosphorylated histone H2AX. Figure 4D shows that the hit compounds exhibited relatively minimal levels of DNA damage when compared to the conventional chemotherapy drug, doxorubicin.

## Discussion

While the past two decades witnessed considerable efforts to target receptors and kinases to impede tumour growth [1, 2, 3, 4, 5, 11, 12, 13, 14, 15, 16, 17], the clinical success of such therapies has often been hampered by drug resistance and toxicity [6, 7, 8, 9, 10]. Although targeting transcription factors was historically disregarded due to structural complexities and intrinsically disordered regions, recent progress in drug discovery and modern chemistry has reignited interest in targeting such proteins. The ETS family of transcription factors is frequently corrupted in cancers [26], and they lie downstream of the major signalling pathways [62, 63], which are misregulated during tumourigenesis. Thus, molecules that specifically inhibit aberrant ETS function could potentially limit overt toxicity and acquired therapy resistance.

Approaches to identify inhibitors of the activity of either specific ETS factors [36, 38, 41] or ETS fusion proteins [37] have included computational chemistry methods and functional screens, which have yielded candidate molecules that target the ETS consensus DNA‐binding site [38], the ribosomal biogenesis machinery [41] or interactions with an RNA helicase [37]. To explore potential ETS factor ligandability and to identify compounds, which can directly block ETS factor function through inhibiting ETS binding to its consensus DNA‐binding site, we have performed proof‐of‐principle small molecule/fragment‐based screens. Two types of screens have been done: (a) fragment‐based screening, using TINS technology, which selects molecular scaffolds that directly bind to the EDBD; (b) a HTS of lead‐like small molecules to identify compounds capable of blocking EDBD binding to its consensus DNA site. The fragment‐based screen demonstrated that small molecular scaffolds could interact with the EDBD (Figs 1 and 2). The relatively weak affinities of the fragments for the target are insufficient to corrupt ETS factor function at therapeutically meaningful doses. However, an advantage of fragment libraries is their chemical diversity and the relatively low molecular weight of the fragments, which significantly increases the chance of identifying primary hits that can be evolved to lead compounds by structure–activity relationships, which can exhibit pharmacologically favourable characteristics [45, 46]. Indeed, we showed that chemically more complex, bulkier analogues of fragment hits could inhibit EDBD DNA binding at concentrations in the range of 100‐fold less than the primary hit candidates. The relative success of the screen measuring ‘direct’ hit–target interactions suggests that comparable approaches might yield ETS inhibitors, such as DNA‐encoded libraries of small molecules, which enable screens on a vast scale (up to billions of compounds), or screens of smaller lead‐like bespoke libraries. The comparative ease of purifying EDBDs coupled to the screening procedure's speed could make this an attractive option [64].

An alternative approach to the ‘direct binder’ screens are functional screens, in this instance, blocking specific binding of EDBDs to a consensus DNA site. HTRF assays are rapid and scalable for screening small molecule libraries (> 400 000 molecules). In our study, such an approach yielded compounds, which could disrupt EDBD binding to DNA and inhibit the proliferation of tumour cells (which harbour illicitly activated ETS proteins). ETS factors such as ETV6, ETS1, FLI1 and ERG are essential for sprouting angiogenesis [31, 32, 33], one of the hallmarks of cancer, and we found that the selected compounds could efficiently inhibit this process, raising the possibility that ETS inhibition might ultimately represent a kill‐two‐birds‐with‐one‐stone approach to tumour targeting. The compounds did not significantly corrupt binding of JUN to its consensus DNA site, consistent with the idea that the compounds displayed specificity. However, they were not selective in inhibiting specific ETS factors and instead blocked DNA binding of multiple different ETS family members (Fig. 3C), which could reflect the high overall amino acid sequence conservation of EDBDs and comparable modes of DNA binding. Of note, the compounds identified in the two different screens do not share obvious structural similarities, which is not surprising since the compound libraries used (fragment library and a library of higher molecular mass small molecules) are evidently distinct. It could also reflect the fact that the screens were also functionally different: the fragment library screen identified molecules, which potentially bind directly to the ‘free’ (unbound) target whilst the multicomponent small molecule screen identified compounds that potentially disrupt EDBD/DNA binding. Thus, the conformation of the EDBD could be different in each case and it cannot be ruled out that compounds in the latter screen interact with a EDBD/DNA complex. Indeed, the lack of obvious EDBD specificity and their relatively high potency in cell‐based assays would be consistent with this view. Generic ETS inhibitors could be of value since they are likely to inhibit tumour cell proliferation more efficiently; however, it is not unlikely that, *in vivo*, such inhibitors might exhibit toxic side effects, due to their role in normal cell signalling networks. Related to this, it is, however, noteworthy, that the compounds were not associated with high levels of DNA damage in cultured cardiomyocytes compared with the commonly used chemotherapy drug, doxorubicin (Fig. 4D). Moreover, zebrafish embryos tolerated low μm quantities of the compounds (Fig. 4).

Are there possible solutions to overcoming the two principal challenges of targeting ETS family members, namely, the identification of specific inhibitors, which exclusively target a single family member, and addressing the issue of low affinities of compounds for their target? A number of approaches could be taken. First, although there are significant technical challenges to surmount, screening libraries against pure, functional full‐length ETS proteins, instead of isolated EDBDs, could enhance specificity, particularly if hit compounds are counter‐screened against other purified ETS family members. Second, and coupled to this, significant advances in covalent library construction, diversity and screening methods [65] could greatly improve compound affinities since they bind irreversibly to their target.

## Author contributions

SA, ZF, JGH, RN and DS performed the experiments. JM performed zebrafish xenotransplants. PD, KS and PCWH had advisory roles. DAB supervised the project and wrote the paper with SA, ZF and DS. All authors read and approved the manuscript.

## Peer review

The peer review history for this article is available at https://www.webofscience.com/api/gateway/wos/peer‐review/10.1002/1873‐3468.70040.

## Data Availability

All the primary data are available upon request to the corresponding authors.
